# Functional diversity buffers the effects of a pulse perturbation on the dynamics of tritrophic food webs

**DOI:** 10.1002/ece3.8214

**Published:** 2021-11-04

**Authors:** Laurie Anne Wojcik, Ruben Ceulemans, Ursula Gaedke

**Affiliations:** ^1^ Ecology and Ecosystem Modelling Group University of Potsdam Potsdam Germany

**Keywords:** functional diversity, nutrient spike, pulse perturbation, regime shift, robustness, tritrophic food web

## Abstract

Biodiversity decline causes a loss of functional diversity, which threatens ecosystems through a dangerous feedback loop: This loss may hamper ecosystems’ ability to buffer environmental changes, leading to further biodiversity losses. In this context, the increasing frequency of human‐induced excessive loading of nutrients causes major problems in aquatic systems. Previous studies investigating how functional diversity influences the response of food webs to disturbances have mainly considered systems with at most two functionally diverse trophic levels. We investigated the effects of functional diversity on the robustness, that is, resistance, resilience, and elasticity, using a tritrophic—and thus more realistic—plankton food web model. We compared a non‐adaptive food chain with no diversity within the individual trophic levels to a more diverse food web with three adaptive trophic levels. The species fitness differences were balanced through trade‐offs between defense/growth rate for prey and selectivity/half‐saturation constant for predators. We showed that the resistance, resilience, and elasticity of tritrophic food webs decreased with larger perturbation sizes and depended on the state of the system when the perturbation occurred. Importantly, we found that a more diverse food web was generally more resistant and resilient but its elasticity was context‐dependent. Particularly, functional diversity reduced the probability of a regime shift toward a non‐desirable alternative state. The basal‐intermediate interaction consistently determined the robustness against a nutrient pulse despite the complex influence of the shape and type of the dynamical attractors. This relationship was strongly influenced by the diversity present and the third trophic level. Overall, using a food web model of realistic complexity, this study confirms the destructive potential of the positive feedback loop between biodiversity loss and robustness, by uncovering mechanisms leading to a decrease in resistance, resilience, and potentially elasticity as functional diversity declines.

## Graphical abstract

The response of a tritrophic food web to a nutrient pulse depends (1) on the amount of extra nutrients added (the perturbation size *N_p_*) and (2) on the moment at which the perturbation is applied, and in particular on top‐down regulatory processes. Importantly, the system response depends (3) on the functional diversity present. Functional diversity has positive or context‐dependent direct effects (in green) and indirect effects (in violet)—by increasing the probability of the system to be on the ecologically desirable high‐production attractor (HP)—on the robustness, that is, the resistance, resilience, and elasticity.

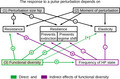

## 1 INTRODUCTION

Human activities undeniably disrupt ecosystem structure and functioning (Cardinale et al., 2012; Hautier et al., 2015; Hooper et al., 2005). Direct effects such as habitat loss due to pollution (Dudgeon et al., 2006; Hölker et al., 2010) and increased land/sea‐use change (Díaz et al., 2019; Ryser et al., 2019) are major causes of the observed losses in biodiversity worldwide. Moreover, climate change effects have a decisive influence on these losses (Bestion et al., 2020): In addition to the global temperature rise (Hansen et al., 2006), the frequency of disruptive extreme weather events has increased steadily (Easterling et al., 2000). For instance, recurrent storms or heavy rainfalls amplify excessive nutrient loading in rivers, lakes, and coastal areas, causing species losses (Øygarden et al., 2014). The combined effect of these processes on biodiversity creates a potentially dangerous feedback loop. When biodiversity is lost, the respective decrease in functional diversity may alter the ecosystem’s ability to buffer perturbations (Cardinale et al., 2012). This leads to additional biodiversity losses and consequently to even more vulnerable ecosystems.

In the last decades, the functional perspective has brought new insights to quantify the effects of biodiversity loss on ecosystem functioning (Tirok & Gaedke, 2010; Violle et al., 2007). One approach entails sorting out the members of a food web into functional groups with similar trait values or to explicitly consider the trait values and their distributions within trophic levels. In this way, morphological, physiological, or behavioral individual characteristics are linked to a certain function, such as growth rate or nutrient uptake (Garnier et al., 2015), and depend on each other by trade‐offs to determine the overall fitness (McGill et al., 2006; Violle et al., 2007). This approach makes explicit how trait changes can feed back to population and food web dynamics, and partly regulate the response of food webs to environmental changes (Raatz et al., 2019; Theodosiou et al., 2019; Yamamichi & Miner, 2015).

Most studies investigating the responses of food webs to perturbations are restricted to multitrophic systems where only one or two trophic levels may adapt (Kovach‐Orr & Fussmann, 2013; Persson et al., 2001), or to strictly bitrophic systems (Fussmann & Gonzalez, 2013; Raatz et al., 2019; Yamamichi & Miner, 2015). These studies underline how functional diversity at one or two trophic levels generally enhances the ecosystem's ability to buffer against perturbations, and that the effects of functional diversity can be modulated at different trophic levels. However, tritrophic systems with at least two functionally diverse trophic levels are more realistic, since strictly bitrophic interactions are rare in nature (Abdala‐Roberts et al., 2019; Matsuno & Nobuaki, 1996), and top predators can have a large influence on ecosystem functioning and dynamics (Ceulemans et al., 2021; Estes et al., 2011).

To properly understand how tritrophic food webs respond to environmental changes, insights are needed into the mechanisms driving their response. External disturbances come in a variety of forms, and each can affect the food web and its functions in different ways. Broadly, external disturbances can be separated into two types, called press and pulse perturbations (Oliver et al., 2015; Raatz et al., 2019). Press perturbations are long‐term or permanent changes to a system component, such as increased harvesting or warming. In contrast, pulse perturbations are short‐term and quasi‐instantaneous changes to state variables, such as species biomasses or nutrient concentration, for example, due to a forest fire or massive rainfall causing heavy run‐off (Bender et al., 1984; Harris et al., 2018).

In this study, we investigated the effects of a nutrient pulse on the dynamics of tritrophic food webs with different levels of functional diversity. Nutrient pulses correspond to a temporary boost of the habitat productivity, which can destabilize the dynamics of food webs and put species at increased risk of extinction (Rosenzweig, 1971). In aquatic systems, such events are happening with increased frequency and magnitude (Galloway et al., 2008; Kaushal et al., 2014). This is highly worrying, because eutrophication leads to a drastic reduction in water quality (Couture et al., 2018; Díaz et al., 2019), to the appearance of anoxic dead zones (Diaz & Rosenberg, 2008), and to changes in the usual seasonal pattern of phytoplankton and zooplankton (Sommer et al., 2012). Importantly, by affecting the timing and the amplitude of plankton biomass peaks, the dynamics of the upper trophic levels may be strongly affected as well (Cloern & Jassby, 2008). Moreover, sudden increases in available nutrients can lead to changes in ecosystem functions, which may be difficult to reverse (Carpenter, 2005). The probability of such a regime shift may be influenced by the amount of diversity present in the ecosystem (Ceulemans et al., 2019; Folke et al., 2004), but explicit demonstration of the mechanisms by which this happens remains difficult.

The response of a tritrophic food web to a nutrient pulse is characterized by several aspects, which are measured by different quantities. Analogous to Grimm and Wissel (1997) and Raatz et al. (2019), the following terms are used:

- Resistance refers to the maximum temporary change in dynamics after a pulse perturbation.
- Resilience refers to whether or not the system returns to its original state after a pulse perturbation.
- Elasticity refers to how quickly the system returns to its original state.

These three quantities are evaluated by several properties of the food web dynamics. The resistance is evaluated shortly after the perturbation. When the dynamics are strongly affected before the system returns to its original state, the resistance is low. The resilience is determined by examining the dynamics after a long time period following the perturbation. If the system does not return to its original state, it is not resilient. Finally, the elasticity is estimated through the return time, which is the time it takes for the system to return to the original state. A lower return time corresponds to a higher elasticity. In this study, robustness is a catch‐all term for designating resistance, resilience, and elasticity.

**FIGURE 1:**
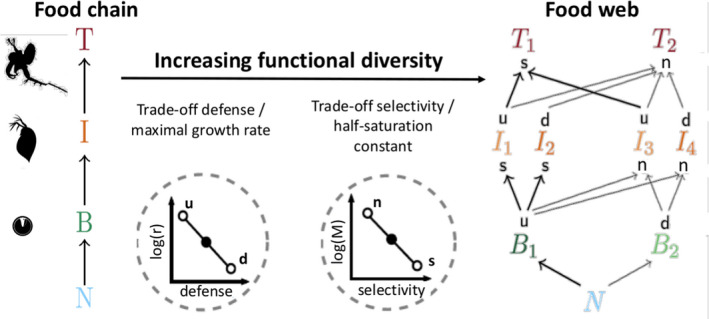
Comparison of two food webs with different functional diversity. The system with no functional diversity (left side, “chain”) is a non‐adaptive food chain, where nutrients (N) are taken up by a basal species (B), which is consumed by an intermediate species (I), which is preyed on by a top species (T). In the most diverse system (right side, “web”), prey species are either undefended (*u*) or defended (*d*), and predators are either selective (*s*) or non‐selective (*n*) feeder. The basal and the top species each differ in one trait, whereas the intermediate species, being both consumers and prey, differ in two traits, resulting in four functionally unique species. Two trade‐offs are used to balance the fitness of the species: A higher defense comes at the cost of a lower growth rate (r), and being less selective implies a larger prey spectrum, but also an increased prey uptake half‐saturation constant (M). In this way, a defended species grows slower than an undefended one and a selective feeder can more efficiently exploit low prey concentrations. The resulting differences in trophic interaction strengths are shown by the arrow thickness between the species (see Appendix 1: A1 for more details about the equivalence of a 4D chain and a 9D web with no functional diversity)

We investigated the response of tritrophic food webs with different levels of functional diversity to a temporal nutrient increase, by focusing on a comparison between a tritrophic non‐adaptive food chain and a food web which was adaptive at each trophic level. We used a food web model, which was adaptive in the sense of species sorting, as described in Ceulemans et al. (2019). Prey species were either defended or undefended, and predator species were either selective or non‐selective feeder (Figure 1). Their relative importance changed according to ambient conditions, leading to continual changes in the mean trait values at each trophic level. In the previous study, the model was used to investigate the effect of gradual changes in functional diversity on ecosystem functions such as biomass production and variability at different trophic levels, as well as the efficiency of resource use and of energy transfer toward higher trophic levels. The resilience was only briefly inferred from the attractor analysis, and no perturbation was explicitly studied. Here, we hypothesize that the food web response depends on (i) the perturbation size, (ii) the time at which the perturbation occurs, and (iii) on the functional diversity. The last hypothesis implies that a functionally more diverse food web is less affected by a nutrient pulse than a functionally less diverse food web. To enlarge generality and accurately capture the complex behavior of the system, we studied this response in different parameter regions with multiple attractors by varying the Hill exponent of the functional responses.

## 2 METHODS

As a basis for our study, we used the chemostat tritrophic model described in Ceulemans et al. (2019), consisting of free inorganic nutrients (nitrogen, *N*) and three trophic levels: basal (*B*), intermediate (*I*), and top (*T*). In this model, the functional diversity of the food web could be manipulated by changing the trait differences between the species on all trophic levels (Δ); for example, a higher Δ value implies that species within the same functional group become more dissimilar. We focused mostly on two distinct cases: a non‐adaptive food chain with no functional diversity (Δ=0) and the most diverse food web (Δ=1), where all the trophic levels were adaptive in the sense of species sorting (see Figure 1). Note that for this model, a food web where all the species within each trophic level have identical traits and biomasses is equivalent to a food chain (Moisset de Espanés et al., 2021). More details are available in Appendix 1: A1. For convenience, we will henceforth call the food web with no functional diversity “chain” (Δ=0) and the most diverse food web “web” (Δ=1).

Species have fixed traits and adaptation occurs through the different responses of the asexually reproducing species, altering their share to the total biomass of the respective trophic level and thus also their mean trait value. To reveal the impact of functional diversity in more detail, we also present results for some food webs with intermediate levels of functional diversity by setting Δ between 0 and 1. In the food webs with functional diversity, the species’ fitness varied with the trait difference and arose from two trade‐offs. Prey species were defended (*d*) or undefended (*u*) against predation, but defense came at the cost of a slower growth rate (*r*). Predator species were selective (*s*) or non‐selective (*n*). Selectivity implied preferential feeding on undefended species, and more efficient exploitation of low resource biomass densities due to a lower half‐saturation constant (*M* for *I* and μ for *T*, cf. Equations 4 and 5). Note that we assumed linear trade‐offs for simplicity, even though other trade‐off shapes exist and may affect coexistence (Ehrlich et al., 2017). While in nature, trade‐offs may not be as clearly expressed as assumed in the model, numerous other mechanisms exist in natural systems which can promote coexistence (such as spatial heterogeneity, metacommunities, other density‐dependent effects arising from natural enemies, e.g., pathogens and other predators; see Edwards et al. (2011) and Adler et al. (2013)). Thus, the trade‐offs promoting coexistence built into the model were assumed to represent several mechanisms acting in nature which were not explicitly considered here.

The chain had three species (one per trophic level), and the web had eight species: two basal species, four intermediate species, and two top species (Figure 1). The intermediate trophic level had four species since they were both prey and predator species; therefore, they had two distinct traits, which result in four possible combinations. In all equations, we used these equivalences:

(1) $$B^{u}\equiv B_{1},\;B^{d}\equiv B_{2},\;I_{s}^{u}\equiv I_{1},\;I_{s}^{d}\equiv I_{2},\;I_{n}^{u}\equiv I_{3},\;I_{n}^{d}\equiv I_{4},\;T_{s}\equiv T_{1},\;T_{n}\equiv T_{2}$$

The tritrophic model described in Ceulemans et al. (2019) is written as follows:

(2) $$\left(\begin{array}{c} \dot{N}=\delta \left(N_{0}‐N\right)‐\frac{c_{N}}{c_{C}}\sum_{i}r_{i}B_{i}\\ \dot{B}=r_{i}B_{i}‐\sum_{j}g_{\mathrm{ji}}I_{j}‐\delta B_{i}\\ \dot{I}=e\sum_{j}g_{\mathrm{ji}}I_{j}‐\sum_{i}\gamma_{\mathrm{ij}}T_{i}‐\delta I_{j}\\ \dot{T}=e\sum_{i}\gamma_{\mathrm{ij}}T_{i}‐\delta T_{i} \end{array}\right.$$

In these equations, the species are distinguished by $i\in \left\{1,2\right\}$ at the basal and top trophic level and $j\in \left\{1,2,3,4\right\}$ at the intermediate trophic level. To increase realism, we assumed a continuous in‐ and outflow of nutrients and organisms from our system, reflecting an ongoing decay and nutrient recycling. Such flow‐through systems are experimentally realized using chemostats rather than batch cultures (i.e., isolated culture vessels). The flow‐through rate is determined by the dilution rate δ, which implies a constant density‐independent mortality rate of all organisms. This rate is most relevant for the top predator, given its lower metabolic rate compared to the intermediate and basal species. We assumed nitrogen to be the limiting nutrient (N), with incoming concentration $N_{0}$. Since the nutrients were measured in nitrogen concentration and the species biomass in carbon biomass, the nitrogen‐to‐carbon weight ratio ($c_{N}/c_{C}$) scaled the basal ($B_{i}$) growth terms in the nutrient equation. The following equations give the expressions of the basal growth rate $r_{i}$ and of the basal‐intermediate and intermediate‐top functional responses $g_{\mathrm{ji}}$ and $\gamma_{\mathrm{ij}}$, respectively.

(3) $$r_{i}=r_{i}^{\prime }\frac{N}{N+h_{N}}$$

(4) $$g_{\mathrm{ji}}=g_{j}^{\prime }\frac{{\left(1/M_{\mathrm{ji}}B_{i}\right)}^{h}}{\sum_{i^{\prime }}{\left(1/M_{ji^{\prime }}B_{i^{\prime }}\right)}^{h}+1}$$

(5) $$\gamma_{\mathrm{ij}}=\gamma_{i}^{\prime }\frac{{\left(1/\mu_{\mathrm{ij}}I_{j}\right)}^{h}}{\sum_{j^{\prime }}{\left(1/\mu_{ij^{\prime }}I_{j^{\prime }}\right)}^{h}+1}$$

where $r^{\prime }$ denotes the maximal basal growth rate and $h_{N}$ the nutrient uptake half‐saturation constant; $g^{\prime }$ and $\gamma^{\prime }$ denote the maximal grazing rates of I and T, M and μ the half‐saturation constants of the B‐I and I‐T interaction, and h the Hill exponent.

The two trade‐offs affected the trait values in the above equations. For the prey, the basal species had a growth rate $r_{c}^{\prime }\approx 0.81/\text{day}$ in the chain, whereas in the web, the undefended basal species’ growth rate $r_{u}^{\prime }$ was set to 1/day and for the defended species $r_{d}^{\prime }=0.66/\text{day}$. For the predators, the intermediate and top species in the chain could exploit their prey with a half‐saturation constant $M_{c}=\mu_{c}\approx 424\;\mu g\;\text{C/L}$, respectively. For the web, the intermediate and top selective feeders had half‐saturation constants set to $M_{s}=\mu_{s}=300\;\mu g\;\text{C/L}$ for the undefended prey, and the corresponding value for the non‐selective feeders on all prey types was $M_{n}=\mu_{n}=600\;\mu g\;\text{C/L}$ (See Appendix 1: A1.1, for all the parameter values with units, which reflect a typical plankton food web; detailed legitimations are provided in Ceulemans et al., 2019).

Importantly, our previous study showed that the Hill exponents of the functional responses of the B‐I and I‐T interaction (h, cf. Equations 4 and 5) played an important role in determining the nature of the dynamical attractor on which the system settled (Ceulemans et al., 2019). In particular, two attractors existed for both the chain and the web. One attractor was characterized by large biomass oscillations and a low top biomass. It therefore had a low top total biomass production (proportional to the biomass given the constant mortality rate δ of the top species) and is thus called “low‐production state” (LP). In contrast, the other attractor had small biomass oscillations, a high nutrient exploitation efficiency, and a high top biomass production. It is subsequently referred to as “high‐production state” (HP). In some cases, the chain and the web exhibited bistability (see Table 1) and were potentially under the threat of a regime shift following a perturbation. In our model, we changed the Hill exponent to capture the different dynamical patterns and to consider all possible behaviors of the model after the perturbation. The Hill exponent was kept above 1.05 to make sure that all the species coexisted. In particular, we selected three values of Hill exponents (h=1.05, h=1.10, and h=1.15) for which we investigated the system's response.

**TABLE 1:** Summary of the effect of the three values of Hill exponent *h* on the attractors found in the chain, web, and systems with intermediate levels of functional diversity Δ=0.33 and Δ=0.66

h	Attractor shape and type			
	Chain Δ=0	Δ=0.33	Δ=0.66	web Δ=1
1.05	LP: limit cycle	**LP: limit cycle**	**LP: limit cycle**	**LP: chaotic**
		HP: limit cycle	HP: limit cycle	HP: limit cycle
1.10	**LP: limit cycle**	HP: limit cycle	HP: fixed point	HP: limit cycle
	HP: limit cycle			
1.15	HP: limit cycle	HP: fixed point	HP: fixed point	HP: fixed point

Note

**FIGURE A4:**
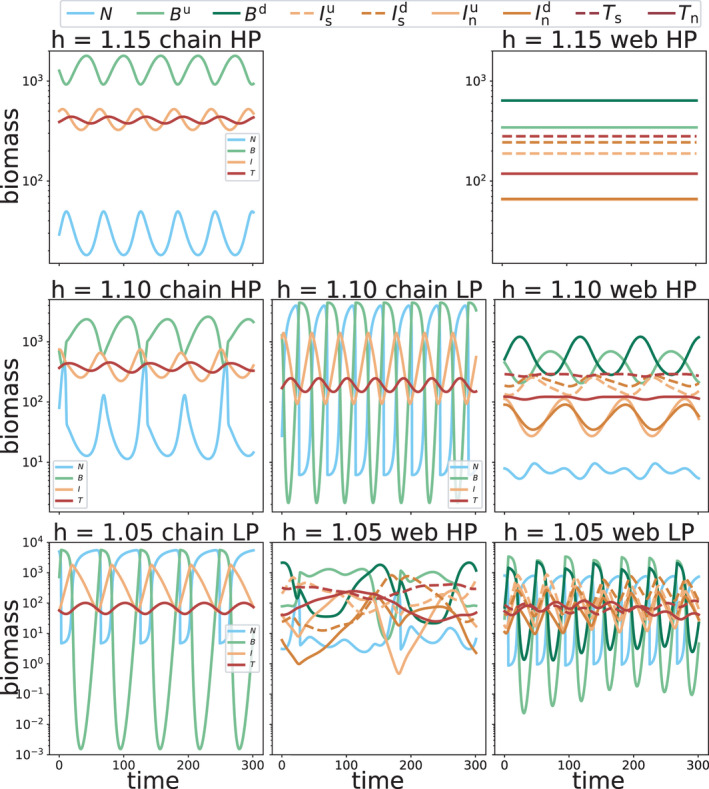
Short overview of the dynamics on each of the different attractors. The type and shape of the attractors depend on the Hill exponent h (cf. Table 1). Note the different biomass scales per row. The top legend only applies to panels in which the dynamics of the food web are shown. Notice how the high‐production (HP) attractor is characterized by a low mean free nutrient concentration, high top biomass, and generally low temporal variability, in contrast to the low‐production (LP) attractor

The system either settles on the low‐production state (LP), the high‐production state (HP), or is bistable. In case of bistability, the more likely state to observe appears in bold characters. A visual representation of the dynamics on each attractor of the chain and the web is given in Figure A4. In this text, we distinguish between attractor *type*, denoting whether the attractor is a fixed point, limit cycle, or chaotic, and attractor *shape*, distinguishing between the HP and LP states. By varying the Hill exponent *h*, we can investigate the effect of a nutrient pulse perturbation under different dynamical regimes.

**FIGURE 2:**
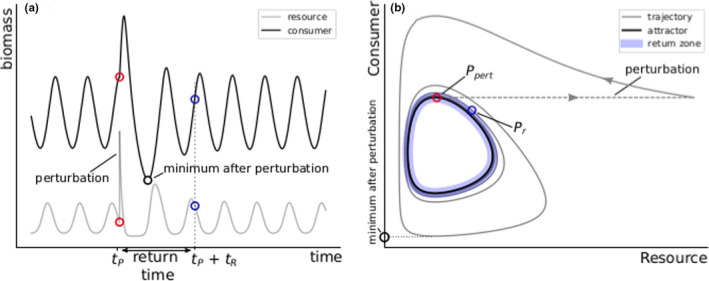
Schematic graphical examples of the different quantities calculated for our results. To study the ecosystem response to a perturbation, we recorded the minimal biomasses reached by the populations after the perturbation, as well as the time it took for the trajectory to return to the vicinity of the attractor. Panel (a) shows the timeseries of a simple consumer‐resource model. The system is oscillating on its stable limit cycle until a nutrient pulse perturbation is applied at time $t_{P}$ (red circle), after which the consumer increases and then declines to very low values. Panel (b) shows the same timeseries data but plotted in phase space. The limit cycle on which the system is oscillating originally is shown by the black curve. The nutrient pulse perturbation is applied at the red point $P_{\text{pert}}$ (also shown in panel (a)), after which the system settles back on the attractor. The return time $t_{R}$ is measured by the time it takes for the trajectory to remain inside the close neighborhood of the attractor (return zone, indicated by the colored region). This happens at the blue point $P_{r}$ (also indicated in panel (a))

To evaluate the system's response to a nutrient pulse, we always ensured that the system was at an attractor (Figure 2), and not still in a transient state, that is, its solution did not change anymore when extending the simulation time. Importantly, the attractor may have a more complex structure—called attractor type in the following—than a simple fixed point: It can be a limit cycle or a chaotic attractor. In the latter cases, the individual populations did not settle down to a fixed value but remained oscillating perpetually. When the system settled on the attractor, the perturbation was applied at time $t_{P}$ by altering the free nutrient concentration N:

(6) $$N\to N+N_{P},$$

where $N_{P}$ is the amount of added extra nutrients, also referred to as the perturbation size. This instantaneous change in the state variable N moved the system from its former location on the attractor to a point farther away from it (Figure 2).

After the perturbation, there were three possible outcomes for the perturbed system. The first option was that the pulse perturbation temporarily disturbed the system, by altering its dynamics during a transient phase in which the system settled back on the attractor. In this case, we recorded the time it took for the system to return to the attractor (return time $t_{R}$), as well as the biomass minima and maxima of each population and each trophic level as a whole during this phase (see also Figure 2). Note that the return time could not be calculated whether the attractor showed a chaotic behavior. A second outcome may occur when at least one population biomass reached such a low value that it crossed the extinction threshold set to $10^{‐9}\;\mu g\;\text{C/L}$. Below this value, the population was set to 0 and considered extinct. Consequently, the system could never return to the initial attractor. Such a threshold prevented numerical problems that could occur when state variables reached values extremely close to 0. Third, in bistable systems the trajectory could be pushed inside the other attractor's basin of attraction. Therefore, the system also never returned to the initial system, but all populations were still present in the food web.

Additionally, when the attractor was more complex than a fixed point (i.e., a limit cycle or chaotic attractor), we investigated how the effect of the perturbation on the dynamics depended on where on the attractor it was applied. This means that for each point on the attractor, we perturbed the system and calculated the biomasses’ minima and maxima as well as the return time following this perturbation. For this, we sampled the different attractors in a high spatial resolution. This was achieved in multiple steps. First, starting from an initial condition known to be in the basin of attraction of the relevant attractor, the system was allowed to settle for $10^{5}$ time units using a large timestep $\Delta_{t}=10^{‐1}$ such that a point sufficiently close to the attractor could be obtained. If the attractor was a limit cycle, the system was further integrated for approximately one period using a high temporal resolution ($\Delta_{t}=10^{‐3}$). This created a set of points $S_{t}$ on the attractor. Finally, to sample the attractor in a way such that the distances between the sampled points on the attractor did not become very large when the dynamics were moving very fast, the attractor was interpolated and resampled such that the arc length between consecutive sampled points was equal to 1 (in units of biomass). For this set of spatially sampled points $S_{x}$, the distance between a point on the attractor and the closest point to it in $S_{x}$ was guaranteed to be smaller than 1μgC/L. In our results, we defined the return zone (cf. Figure 2) as the volume in phase space inside which the distance to any point on the attractor was less than 5μgC/L. When the attractor was chaotic, even when performing this procedure over multiple periods, the return time could not be calculated using this method. Hence, we excluded results presenting chaotic attractors in the elasticity analysis.

Ordinary differential equations were solved numerically in C using the SUNDIALS CVODE solver (Hindmarsh et al., 2005) with relative and absolute tolerances set to $10^{‐10}$. Output results were analyzed in Python using several packages among which NumPy, SciPy, and Matplotlib (Hunter, 2007; Van Der Walt et al., 2011).

## 3 RESULTS

**FIGURE 3:**
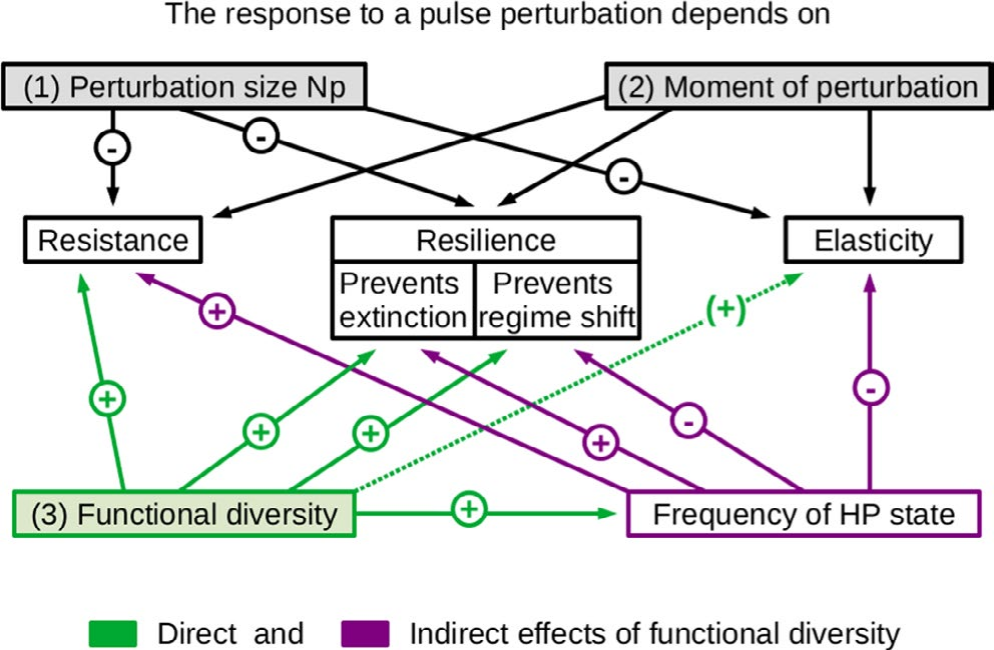
Schematic summary of the response of food webs to a nutrient pulse. This response depends on (1) the amount of extra nutrients added (the perturbation size $N_{p}$) and (2) on the moment at which the perturbation is applied (i.e., the position on the attractor)—and in particular with the ability of the intermediate species to keep the basal species under top‐down control. Importantly, the system response also depends on (3) the functional diversity present. Functional diversity has positive or context‐dependent (see functional diversity–elasticity relationship) direct effects (in green) and indirect effects (in violet)—by increasing the probability to be on the high‐production attractor HP—on the resistance, resilience, and elasticity

We compared the response of a tritrophic non‐diverse chain (“chain,” Δ=0) and a more diverse food web (“web,” Δ=1) to a nutrient pulse, by quantifying its robustness, that is, its resistance, resilience, and elasticity (see Figures 1 and 2). In some cases, we also compared the two above systems to food webs with intermediate levels of functional diversity (0<Δ<1) to generalize our findings. The major results are summarized schematically in Figure 3. Alongside this analysis, we investigated the actual timeseries in detail in order to uncover the mechanisms responsible for the observed responses. Note that for generalizing results we explored several behaviors of the system that we call attractor type and shape (see Table 1). The type characterizes whether the attractor was a fixed point, a limit cycle, or a chaotic. The shape distinguishes between the low‐production attractor (LP)—defined by large biomass oscillations and a low top biomass—and at the opposite the high‐production attractor (HP).

### 3.1 Resistance

**FIGURE 4:**
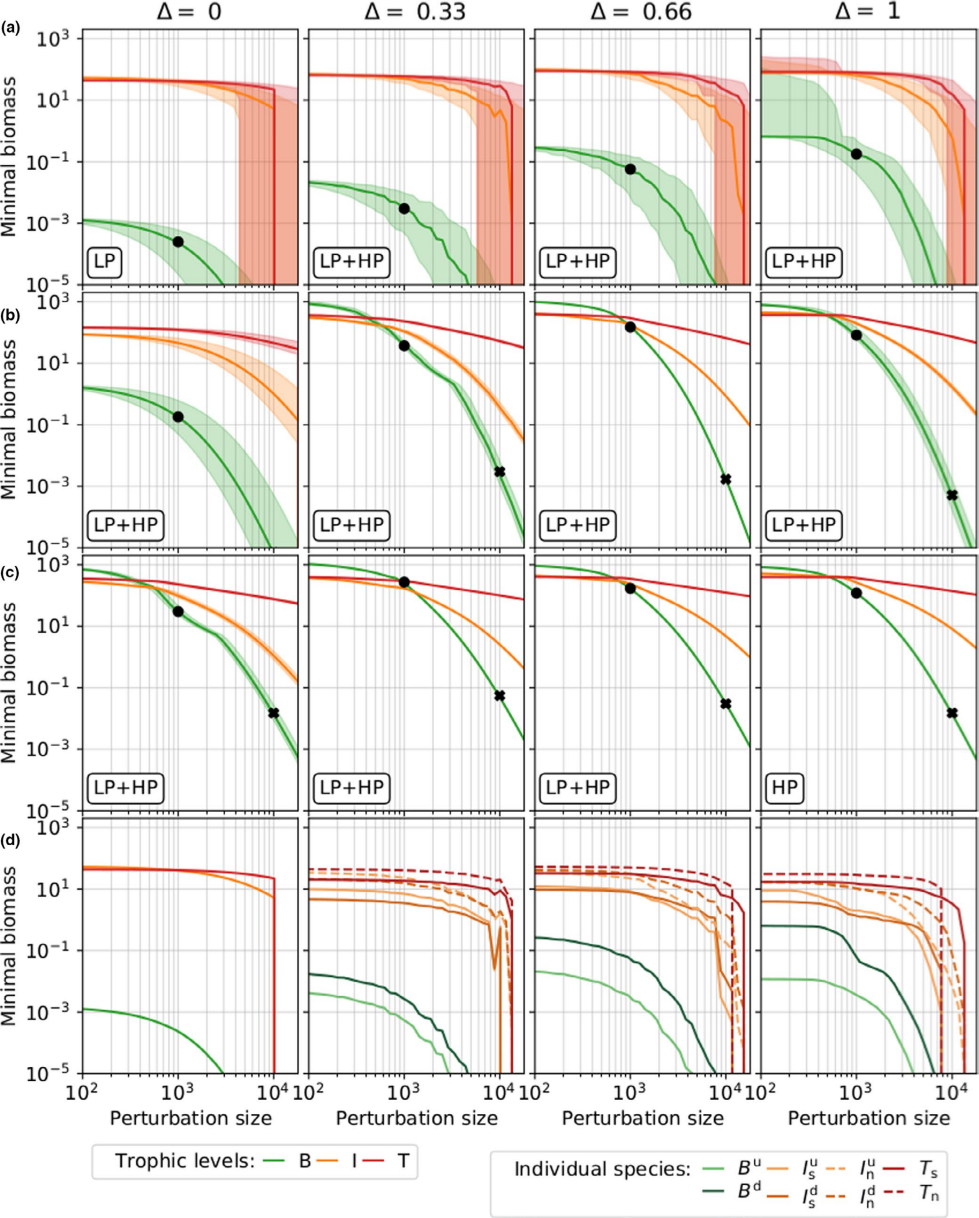
Biomass minima reached by the timeseries after the perturbation as a function of the perturbation size $N_{p}$ for food webs differing in functional diversity (Δ) and Hill exponents. Functional diversity increases from the left to the right columns. Note that Δ=0 is the chain, Δ=1 is the most diverse web (called web in the main text). From row (a) to (c), the Hill exponent increases from h=1.05 to h=1.10 and h=1.15, respectively. Black dots and black crosses mark the minimal biomasses for the basal trophic level reached after a perturbation size of $N_{p}=10^{3}$ and $N_{p}=10^{4}$ μg N/L. Labels in the left‐bottom corner indicate the attractor shape, that is, if the system is on the LP state, HP state, or bistable (LP + HP) before the perturbation (see also 1). Row (d) represents the individual minimal biomasses reached by each species at h=1.05. Note that for the chain (Δ=0) there exist only three species with distinct functional traits. Each line corresponds to the median of 1000 simulations, and the shaded areas are showing the upper and lower quartiles. When shaded areas are not visible in the upper panels (rows a, b, c), the system settled on a fixed point. The quartiles are not displayed in row (d) to increase readability. The patterns shown for minimal biomasses are also observed for maximal biomasses but are quantitatively weaker (see Figure A5)

**FIGURE A5:**
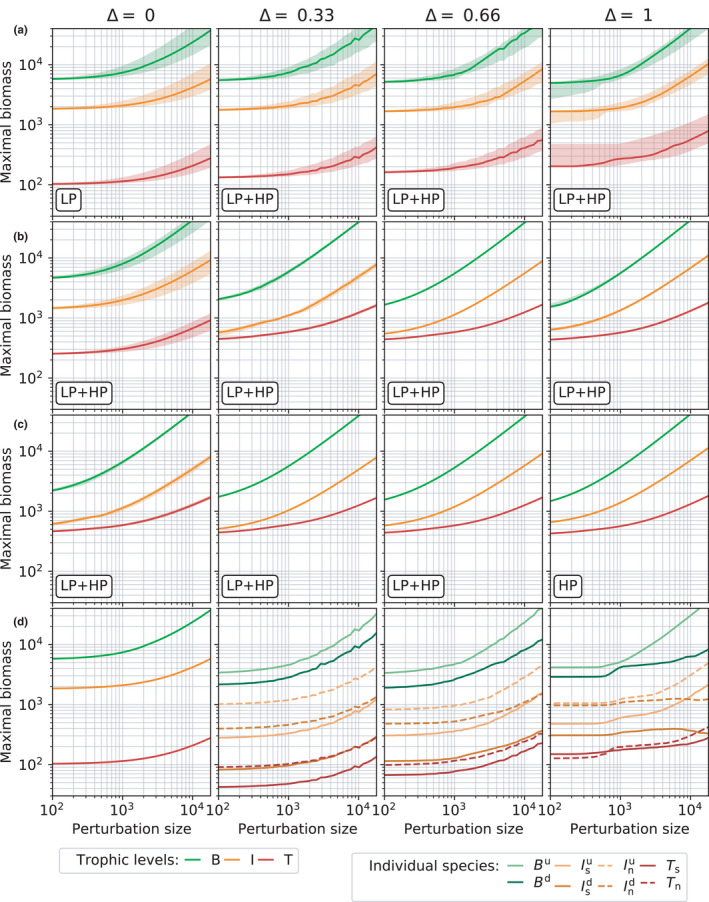
Biomass maxima reached by the timeseries after the perturbation as a function of the perturbation size $N_{p}$ for food webs differing in functional diversity (Δ) and Hill exponents h. Functional diversity increases from the left to the right columns. Note that Δ=0 is the chain, Δ=1 is the more diverse web (called web in the main text). From row (a) to (c), the Hill exponent increases from h=1.05 to h=1.10 and h=1.15, respectively. Black dots and black crosses mark the maximal biomasses for the intermediate trophic level reached after a perturbation size of $N_{p}=10^{3}$ µg N/L and $N_{p}=10^{4}$ µg N/L. Labels in the left‐bottom corner indicate the attractor shape, that is, if the system is on the LP state, HP state, or bistable (LP + HP) before the perturbation (see also 1 in the main text). Row (d) represents the individual maximal biomasses reached by each species at h=1.05. Note that for the chain (Δ=0) there exist only three species with distinct functional traits. Each line corresponds to the median of 1000 simulations, and the shaded areas are showing the upper and lower quartiles. When shaded areas are not visible in the upper panels (rows a, b, c), the system settled on a fixed point. The quartiles are not displayed in row (d) to increase readability. The results for the maximal biomasses confirm the patterns observed for the minimal biomasses but are quantitatively weaker

As a general pattern, resistance, here quantified by the biomass minima reached after a perturbation (cf. Figure 2), tended to decrease as the perturbation size $N_{P}$ (i.e., the added extra nutrients) increased (Figure 4). This implied that, as the amount of added nutrients increased, the biomass amplitudes immediately after the perturbation increased correspondingly, in a highly nonlinear way (see also Figure A5 showing an increase in the maxima). In all cases, the basal level was the most strongly affected by the nutrient pulse, whereas the top level was the least affected.

By randomly selecting 1000 time points at which the perturbation was applied for each perturbation size, we captured how the system response depended on the state of the system at the moment of perturbation. Importantly, this analysis showed how the attractor type influenced resistance. If the equilibrium state was a fixed point (see systems with Δ≥0.33 at h=1.15, Table 1), the response did not depend on the moment of perturbation. All initial conditions led exactly to the same post‐perturbation minimum, and therefore, the median, upper, and lower quartiles were all equal (Figure 4 row c, Δ≥0.33). On the other hand, when the equilibrium state was a limit cycle or a chaotic attractor, the minimum reached by the timeseries might strongly vary depending on when the perturbation was applied. How much this response varied was reflected by the difference between the upper and lower quartiles.

For a very small perturbation in the food web, the spread of the biomass minima was correspondingly small (Figure 4). However, this spread appeared very large for the web when h=1.05 (Figure 4, row a, Δ=1). This discrepancy could be explained by the presence of the two attractors (cf. Table 1), each with a basin of attraction of approximately equal size. Because the actual timeseries minima differed between the attractors, so did the minima after a small perturbation. Thus, what appeared as a very large range between which the minima were distributed was actually a strongly bimodal distribution centered around the respective minima of each attractor. While the chain for h=1.10 (Figure 4 row b, Δ=0) also exhibited bistability (with both the HP and LP states), the basin of attraction of the HP state was so small that this effect did not significantly impact our results (only one initial condition out of 1000 ended up on the HP state).

**FIGURE 5:**
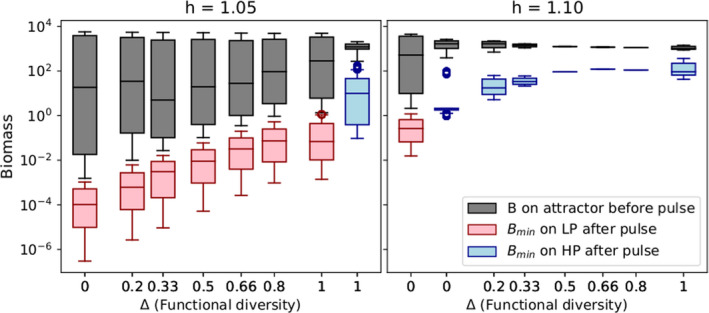
Basal biomasses on the attractor before the perturbation (gray boxplots) and minimal basal biomasses reached after a perturbation of $N_{p}=10^{3}$ µg N/L (red (LP state) and blue (HP state) boxplots depending on the attractor shape) for systems with different levels of functional diversity (i.e., different Δ values). When Δ increases species within each trophic level get more functionally dissimilar, Δ=0 corresponds to the system with no functional diversity (chain) and Δ=1 corresponds to the system with the most dissimilar species (web). Simulations for intermediate values of Δ highlight the gradual positive relationship between functional diversity and the systems resistance for each attractor shape. The left panel shows results for a Hill exponent h=1.05 where most of the systems are on the LP state (for 0<Δ<1, the probability of being on the HP state is <0.2). Note that for Δ=1 there are two boxplots since the web is bistable for this specific Hill exponent (the probability to observe the HP state is non‐negligible—>0.4—and see Table 1). The right panel has a higher Hill exponent h=1.10 for which all the systems are on the HP state except the chain which is bistable. The chain has a low probability to be on the HP state (<0.1) at h=1.10, but this boxplot is displayed to allow the comparison with the other food webs

When comparing the biomass minima reached by each trophic level, we observed a higher resistance when the Hill exponent increased (Figure 4 rows a to c). This pattern held for food webs with different levels of functional diversity and highlighted the importance of the attractor shape (LP or HP state). For the lowest Hill exponent (Figure 4 row a), the LP state was the only or most likely state to exist, whereas for higher Hill exponents, the HP state prevailed except for the chain which was bistable for h=1.10 (see Table 1). For h=1.05 and h=1.10, the web was more resistant than the chain (see the minimal basal biomass reached in the chain and the web after a perturbation size $N_{p}=10^{3}$ µg N/L; Figure 4 black dots and Figure 5). This statement held as well for h=1.15, but in the two first cases, functional diversity was not the only factor influencing systems’ resistance. At h=1.05, resistance increased due to the higher probability of being on the HP state for the web. At h=1.10, the lower resistance of the chain resulted from the coexistence of the LP and HP states, which explained the large increase in the biomass minima from the chain to the other food webs with higher levels of functional diversity. As known from Ceulemans et al. (2019), a higher functional diversity (i.e., a higher Δ) enhanced the probability of being on the HP state. Therefore, one reason explaining why more diverse systems were more resistant is that they were more likely to be on the HP state. For very large perturbation sizes, the effect of functional diversity for systems on the HP state was hardly visible (see the black crosses at $N_{p}=10^{4}$ µg N/L in Figure 4). The results held as well for the maximal biomasses but were quantitatively weaker (Figure A5).

**FIGURE A6:**
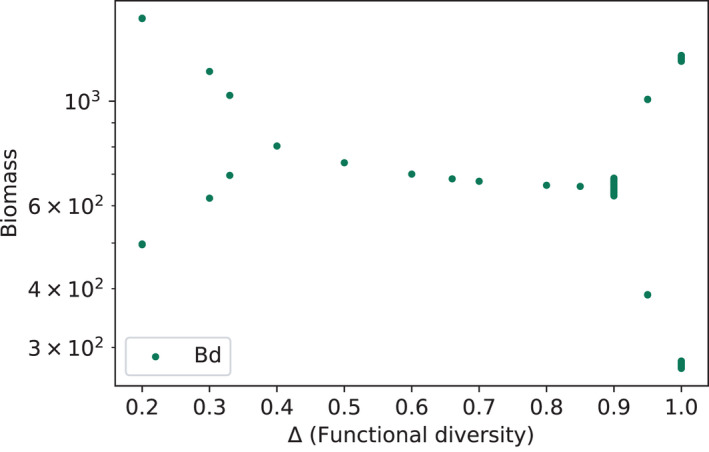
Bifurcation diagram of the defended basal biomass at h=1.10, where only the HP state existed, depending on the functional diversity (Δ). Simulations were run from 1000 different random initial conditions for each value of functional diversity and minimal and maximal biomasses were recorded. Note that below Δ=0.2 the defended basal species did not coexist with the undefended one. By increasing functional diversity, the attractor type changed from a limit cycle to a fixed point, but arriving at high Δ values the attractor type came back to a cyclic behavior. For these high values of Δ, the defended basal species was very dissimilar to the undefended one therefore they had a large niche differentiation and complementary dynamics could be observed

To further assess the relationship between functional diversity and resistance, we investigated this relationship for intermediate levels of functional diversity (0<Δ<1) and separately for the two attractors after a given perturbation size $N_{p}=10^{3}$ µg N/L (see LP state in the left panel and HP state in the right panel of Figure 5). For each attractor, we observed a positive relationship between functional diversity and resistance: With increasing Δ values, the minimal basal biomass reached less extreme values after the pulse perturbation. Note that this relationship was less pronounced for the HP than the LP state and that the most resistant system for this attractor was at Δ=0.66. The small decline in resistance from Δ=0.66 to Δ=1 occurred when the attractor changed from a fixed point to cyclic dynamics (cf. the bifurcation diagram in Figure A6).

**FIGURE 6:**
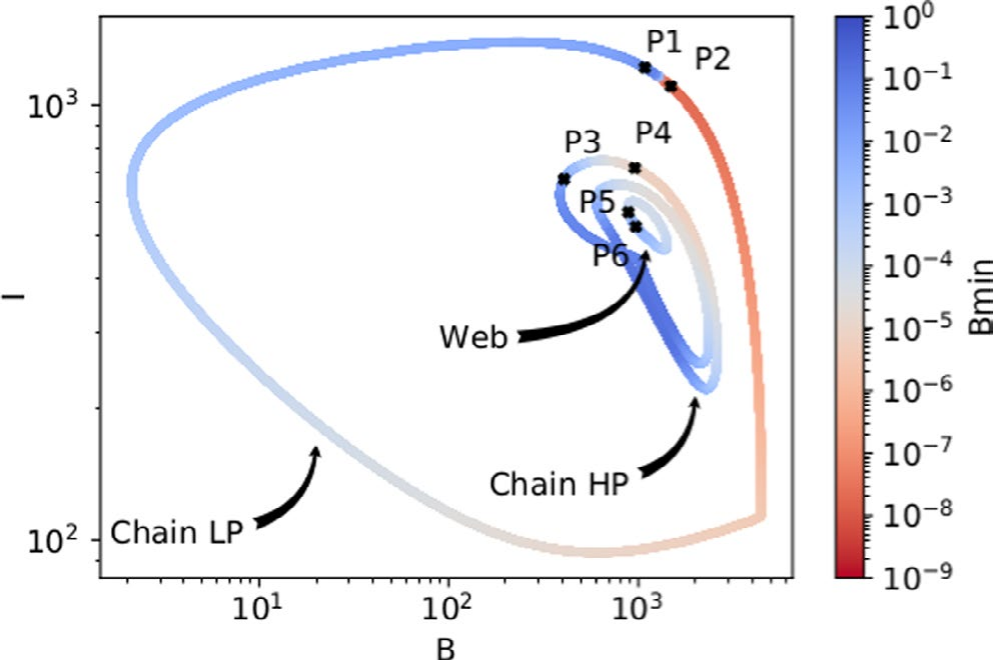
Minima reached by the basal trophic level after the perturbation, depending on where on the attractor a nutrient pulse of size 10^4^ µg N/L is applied, projected on the B‐I plane. The two attractors of the chain (LP, HP), and the HP attractor of the web, when h=1.10, are all shown. The points $P_{1}$ to $P_{6}$ are picked to highlight the large differences in response that are possible by perturbing the system on points that may be very close together. The timeseries of these points are shown in Figure 7. See Figure A7, in the Appendix for all Hill exponents and for different perturbation sizes

**FIGURE 7:**
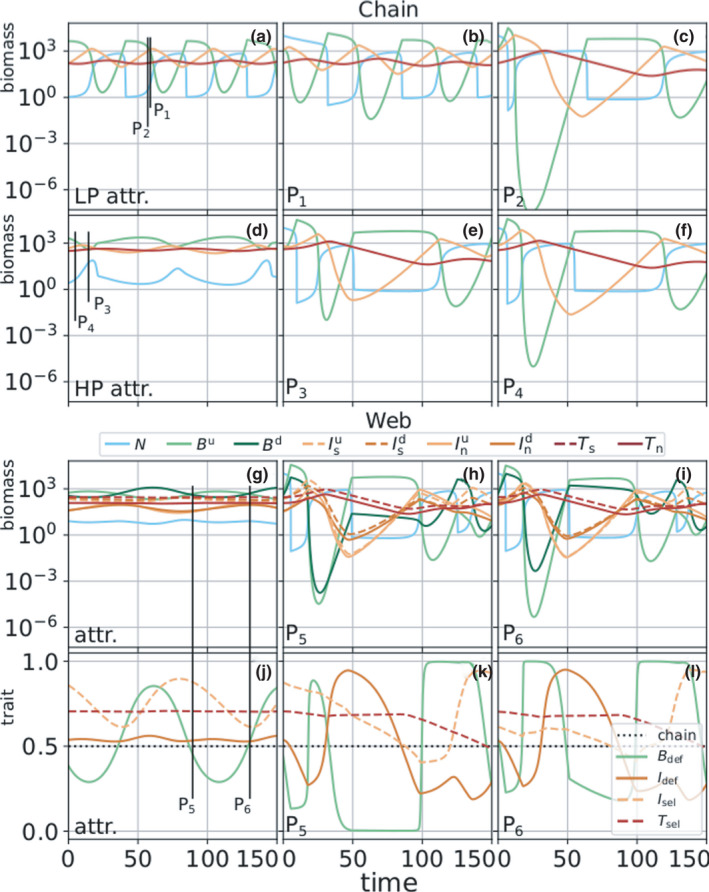
Timeseries of the dynamics of the chain and food web for h=1.10, showing first the dynamics on the attractor, that is, prior to the perturbation (chain LP: panel (a), HP: panel (d), web: panel (g)), and in the middle and right columns the system's behavior after a perturbation of size 10^4^ µg N/L at different moments of perturbation, here indicated by the points $P_{1}$–$P_{6}$ (cf. Figure 6). The locations of these points in time on the attractor are indicated by the vertical black lines in the leftmost column. The bottom row (j–l) shows the temporal development of the trait value for the biomass dynamics shown in the panel above (basal and intermediate defense $B_{\text{def}}\;\text{and}\;I_{\text{def}}$, and intermediate and top selectivity $I_{\text{sel}}\;\text{and}\;T_{\text{sel}}$, cf. Figure 1). The basal defense level $B_{\text{def}}$ is the proportion of the defended basal species $B^{d}$ (cf. Figure 1) of the total amount of basal biomass. The other traits are calculated equivalently. Notably, while $P_{3}$ and $P_{4}$ are on the HP attractor before the perturbation, the extremely high inflow of nutrients pushes the system into the LP state, where it remains. Because the LP state is unstable when h=1.10 in the food web, the trajectories of $P_{5}$ and $P_{6}$ must eventually return to the HP state (cf. Figure A8)

The pronounced differences between the minimal biomass values reached after a perturbation of a given size could be understood by explicitly examining the responses when the perturbation occurred (i.e., along the attractor; see Figure 6 for h=1.10 and $N_{p}=10^{4}$ µg N/L and Figure A4 in Appendix for other *h* and $N_{p}$ values) and the post‐perturbation timeseries (Figure 7). In these figures, we highlighted six points on the attractors and compared the response of the basal trophic level to a large perturbation of size 10^4^ µg N/L (≈10 times the standard nutrient inflow $N_{0}$) applied at each of these points. While $P_{1}$ and $P_{2}$ were very close together on the attractor, the effect of the perturbation on the resulting dynamics was very different between these two points (Figures 6 and 7a–c). In both cases, the basal species were in decline at the moment of the perturbation. At $P_{1}$ (Figure 7b), they were under sufficient top‐down control by the intermediate level, such that the free nutrients could not be efficiently exploited and remained very high for a long period of time. Thus, most of the extra nutrients due to the perturbation were simply washed out of the system. In the case of $P_{2}$ (Figure 7c), however, the basal species were able to exploit almost all of the newly available nutrients immediately. This led to an extremely high peak biomass of the basal level, which was in turn exploited by the intermediate level. Because of the delayed response of the top level, the intermediate species were able to stay at a high biomass for an extended period of time and thus grazed the basal level down to a very low biomass density.

This pattern described what was observed generally in both the chain and the web after a nutrient pulse perturbation. A portion of the supplementary nutrients was quickly taken up by the basal trophic level, which subsequently caused a biomass peak in the intermediate trophic level. In turn, the higher this peak was and the stronger was the grazing pressure on the basal level.

A general negative correlation emerged between the maximal intermediate biomass ($I_{\text{max}}$) and the minimal basal biomass ($B_{\text{min}}$) shortly after the perturbation: The higher the intermediate species grew after the pulse, the more severely they depleted the basal species. Notably, our results showed that a given $I_{\text{max}}$ generally led to a $B_{\text{min}}$ that was approximately one order of magnitude higher in the web, as compared to the chain, and that this effect was not simply due to the different growth and grazing rates, but rather due to the increased functional diversity in the web (cf. Appendix 1: A2 for a detailed explanation). The increase in the biomass of the intermediate trophic level led subsequently to an increase in the biomass of the top trophic level, which, in turn, led to the intermediate level being under strict top‐down control and thus unable to exploit the high basal biomass following the nutrient pulse (cf. Appendix 1: A2; Ceulemans et al. (2019) and Ceulemans et al. (2021)). Thus, the web exhibited stronger top‐down regulatory processes resulting in a higher resistance to a nutrient pulse.

Investigating not only the biomass but also the mean trait dynamics after the perturbation in the web clearly showed how a more diverse food web might be able to buffer the nutrient pulse (Figure 7j–l). Right after the perturbation, the high concentration of available nutrients caused the basal biomass to increase, with the undefended species increasing faster due to its higher growth rate. If the selective intermediate species were sufficiently high in biomass, they were able to graze down the undefended basal species to very low densities, potentially causing the defended basal species to outweigh the undefended species by several orders of magnitude (cf. Figure 7). Importantly, the defended species was not grazed down to such low levels, preventing the potentially very strong reduction in total basal biomass observed in the chain.

Additionally, the effect of the nutrient pulse propagated successively to higher trophic levels (Figure 7j–l). Thus, the trait composition of the top trophic level was only substantially altered long after the perturbation. This second mechanism could only take place in a functionally diverse community: In the linear chain, there was no potential for the mean trait value to adapt to a perturbation and thus no capacity for buffering.

### 3.2 Resilience

Quantifying the resilience of a food web required determining whether the pre‐perturbation and post‐perturbation states differed from each other either when the perturbation resulted in extinction of at least one population, or when the food web settled on a different attractor—on which all species coexisted.

Following a sufficiently large nutrient pulse, the basal trophic level biomass crossed the numerical extinction threshold of 10^−9^ µg C/L first, leading to additional extinctions on the I and T levels (Figure 4). When h=1.10 or 1.15, a significant proportion of extinctions only happened for unrealistically large perturbation sizes outside of the range we considered. However, when h=1.05, this became a likely occurrence starting from perturbation sizes of approximately 3 × 10^3^ µg N/L in the chain, as compared to 7 × 10^3^ µg N/L in the web (Figure 4 Δ=0 and Δ=1 in row a). Importantly, extinctions of individual populations might already happen for smaller perturbation sizes in the web ($\approx 3\times 10^{3}$ µg N/L), and this led to extinctions on higher trophic levels as well. However, the remaining population on the basal level could still support at least a part of the web. In contrast, the extinction of the basal population in the chain invariably led to the complete disappearance of all the upper trophic levels as well. Thus, the web exhibited a higher resilience than the chain.

The above results did not account for potential differences in resilience between different attractors of the system in case of bistability (see Table 1). Previous investigations into this model showed that the basins of attraction of the high‐production (HP) and the low‐production (LP) attractors were significantly influenced by the functional diversity present: The HP attractor was strongly promoted as diversity increased (see previous subsection Resistance and Figure 3), through efficient nutrient exploitation facilitated through compensatory dynamical patterns (Ceulemans et al., 2019).

**FIGURE 8:**
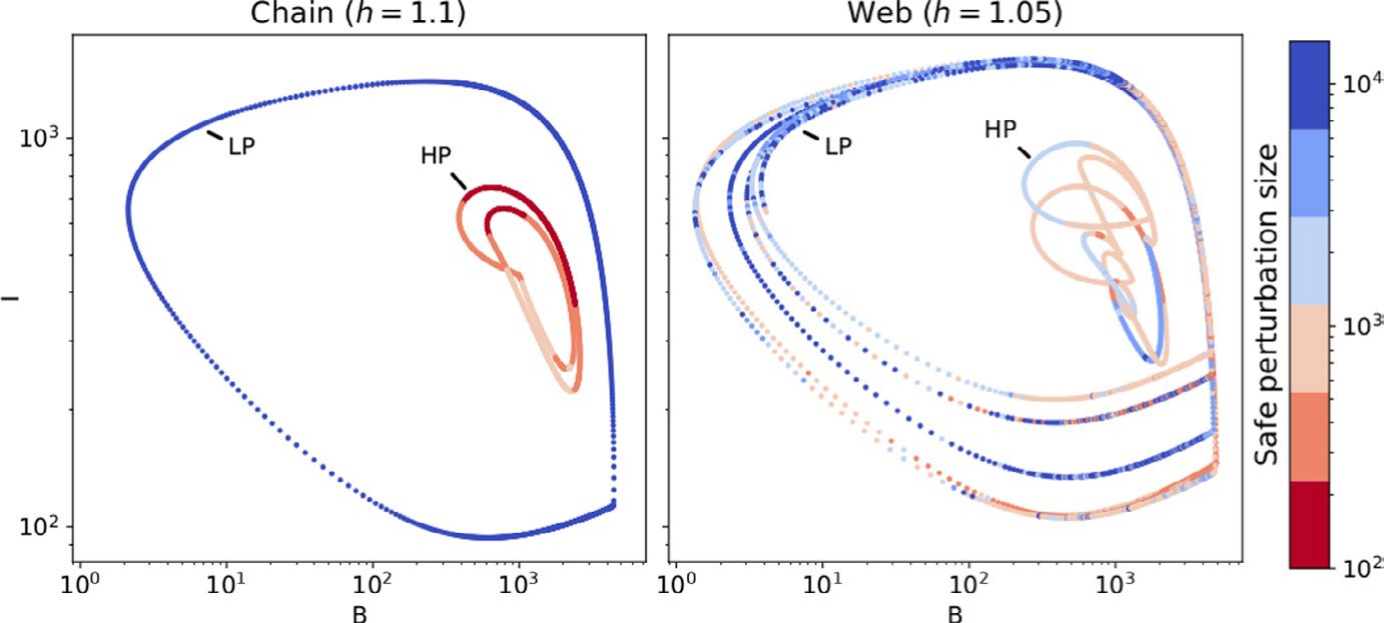
Total basal (B) and intermediate (I) biomass on the low‐production (LP) and high‐production (HP) attractors, for both the chain (left) and web (right) when they exhibit bistability. This happens when the Hill exponent h=1.10 in the chain, and h=1.05 in the web. The color indicates the maximum perturbation size for which the system, when perturbed at this point in the attractor, still returns to its original state. When the perturbation is larger than this “safe” perturbation size, the system either settles on the other attractor, or to another non‐coexistence attractor. On the chain (left), the LP state is very resilient to perturbations, because even for perturbations of size 10^4^ µg N/L, the system returns to this state, independently of where on the attractor it is applied (recall that $N_{0}\approx 1000\;\mu g\;\text{N/L}$). Conversely, the HP state is very vulnerable: A perturbation size of ≈200μgN/L frequently moves the system outside the HP’s basin of attraction, and the maximum safe perturbation is ≈1000μgN/L. For the web, the same pattern is observed: Less points on the HP state are resilient than those on the LP state. However, points on the HP state for the web are much more resilient than those for the chain, despite its lower Hill exponent of h=1.05

When comparing the resilience of the HP and LP attractors to a perturbation separately, we found that the HP state was more vulnerable in both the chain and the web (Figure 8). In the chain, with h=1.10, perturbation sizes of maximally ≈1000μgN/L, but even as small as ≈100μgN/L on the HP state, could move the system outside its basin of attraction, such that it settled on the LP state. In contrast, more points on the LP attractor exhibited resilience: Even after perturbations of size 10,000μgN/L anywhere on the attractor, the system still returned to it (recall that $N_{0}\approx 1000\;\mu g\;\text{N/L}$; cf. Appendix 1: A1.1).

For the web, with h=1.05, the situation was qualitatively similar, but there were some important differences. The LP state still showed resilience to perturbations of ≈10,000μgN/L, but not over its full length. Recall that, when h=1.05, the likelihood of extinctions became non‐negligible for perturbation sizes from ≈3000μgN/L or higher (cf. Figure 4). Furthermore, there were some regions where perturbations of ≈200μgN/L caused a transition from the LP to the HP state. The HP attractor was resilient to perturbation sizes of ≈5000μgN/L for some areas, in contrast to the chain. Points $P_{3}$ and $P_{4}$ (Figure 7d–f) illustrate how, when a perturbation was applied in the HP state, the system's dynamics changed to the LP state, and consequently, returned to the LP state instead of the HP state. Nonetheless, our results showed that functional diversity balanced the transition probabilities between these two states, thereby greatly increasing the resilience of the HP state.

### 3.3 Elasticity

**FIGURE 9:**
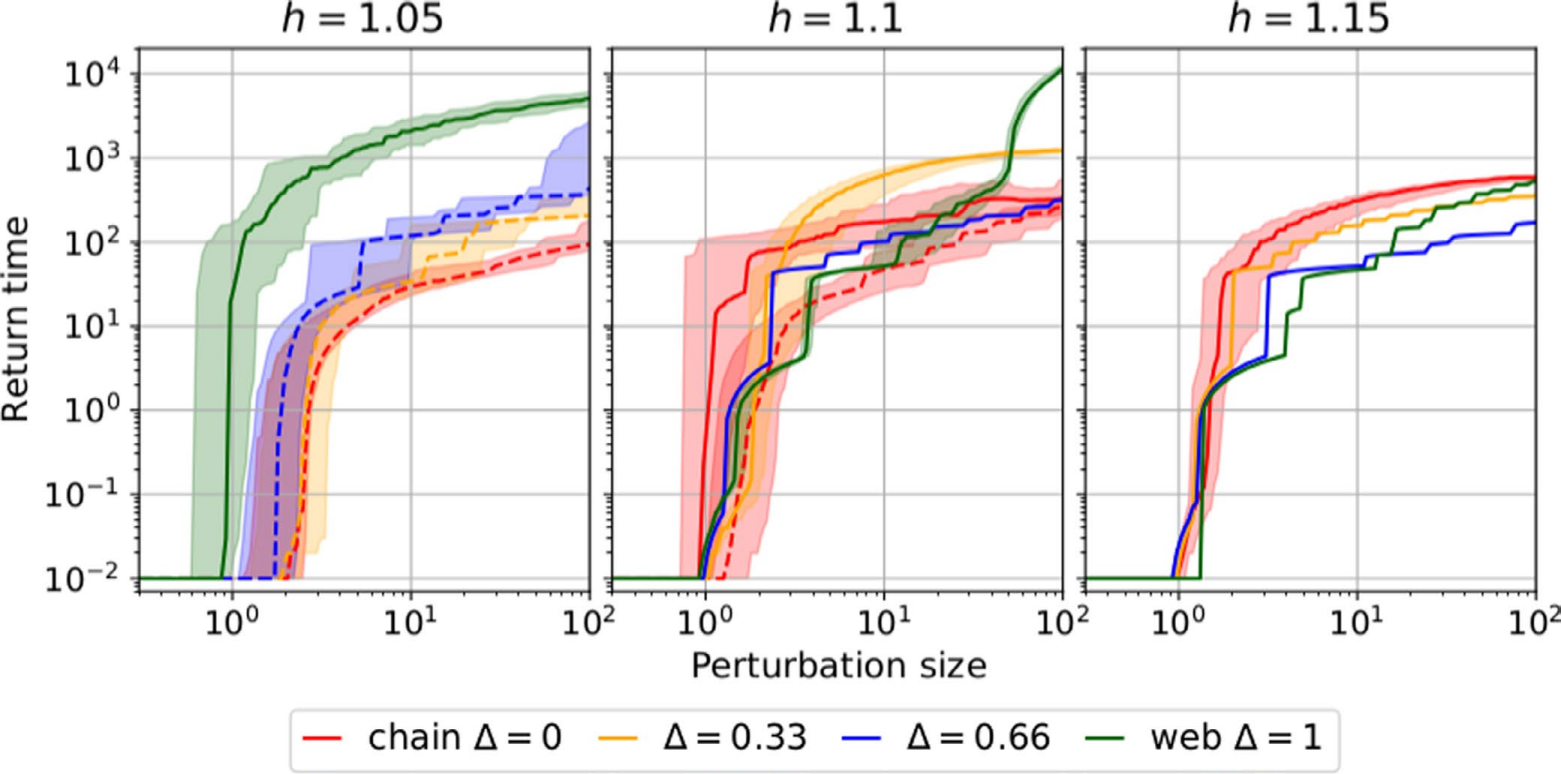
Return time as a function of the perturbation size for the different attractor shapes in our system (LP in dashed line and HP in plain line), for different levels of functional diversity (none/chain Δ=0, low Δ=0.33, intermediate Δ=0.66, and high Δ=1), for three different Hill exponents (*h*). The return time for the LP state in the web Δ=1 when h=1.05 (cf. Table 1) could not be calculated. In all other cases, the median and lower and upper quantiles of the return times for 100 evenly spaced points on the attractor are shown for each perturbation size where applicable (for h=1.15 systems with Δ>0 and for h=1.10 Δ=0.66 the systems settle on a fixed point, cf. Table 1). The minimum return time (10^−2^) is determined by the time step at which the timeseries were sampled. A lower return time means that the system returns faster to its original attractor, implying a higher elasticity. While a straightforward comparison of the elasticity of the chain versus the web proves difficult, our results show that the LP state tends to have a higher elasticity to nutrient pulse perturbations than the HP state. For individual attractor shapes (LP or HP), the functional diversity effect is context‐dependent since it is not consistent across perturbation size ranges and h

To quantify the elasticity in our system, we estimated the median return time as a function of the perturbation size (Figure 9). This is defined as the time required for the system to return and subsequently to stay within the attractor's near vicinity (maximal distance to a point on the attractor must be <5 in units of total biomass) after a perturbation (cf. Figure 2). Because this time might vary depending on where on the attractor the perturbation was applied, the median return time and the lower and upper quantiles of 100 evenly spaced points on the attractor are displayed. The perturbation size was only increased up to 100μgN/L ($\approx 0.1\;N_{0}$) to prevent the influence of trajectories not returning to their original attractor.

Due to the significant structural differences between a chain and a web (Figure 1), a straightforward comparison between the elasticity of these two systems was difficult. An additional complicating factor was the different number and/or type of attractors for systems with different levels of functional diversity at a given Hill exponent (see Table 1). Indeed, if the attractor was a fixed point before the perturbation, its return time was shorter than for a limit cycle. Despite these complicating factors, the return time of the LP attractor tended to be lower than that of the HP attractor; in other words, the LP attractor tended to be more elastic than the HP attractor.

With this effect of the attractor shape in mind, our results showed that the return time was influenced by the level of functional diversity in the system. For h=1.05 (Figure 9, left panel), a higher functional diversity led to a higher return time for systems on the LP state (Δ≤0.66). The web (Δ=1) could not be meaningfully compared since the return time could only be calculated for the HP state. When h=1.10 (Figure 9, middle panel), the effect of functional diversity on the elasticity depended on the perturbation size $N_{p}$. For small perturbations (*N_p_* < 10 µg N/L), more diverse systems had smaller return times. For instance, the return time of the web was approximately an order of magnitude lower than the chain on the HP state. For higher perturbation sizes (*N_p_* > 10 µg N/L), we observed the opposite trend: More diverse systems had higher return times for a same attractor shape (HP). This was due to the different attractor types (fixed point for Δ=0.66 and limit cycles for the other systems; see Table 1) and the varying systems’ complexity, for example, the increased complexity of the web caused the return time to increase faster than it did for the chain. For h=1.15, all systems were on the HP state and were fixed points, except the chain, which was a limit cycle. The return time of the web was roughly an order of magnitude lower than that of the chain. We thus found that, in contrast to the pattern found for h=1.05, elasticity increased with functional diversity (Figure 9, right panel). To conclude, the direction of the effect of functional diversity on elasticity was not consistent across perturbation size ranges and Hill exponents.

## 4 DISCUSSION

We compared the consequences of a perturbation by a nutrient pulse for the dynamics of a non‐diverse and a more diverse tritrophic food web. The non‐diverse food web was a non‐adaptive food chain with three species (“chain,” Δ=0), whereas the most diverse food web (“web,” Δ=1) had three adaptive trophic levels and eight species (Figure 1). In the web, prey species could be defended or undefended against predation, and the consumer species could be selective or non‐selective feeders. Fitness differences were balanced by two trade‐offs: Defended species grew slower, and non‐selective feeders exploited low resource densities less efficiently.

We showed that, in accordance with our first hypothesis, a higher pulse perturbation had stronger effects on the food web dynamics (Figures 3, 4, and 9). Temporarily increasing the available nutrients affected the trophic levels differently in our study, which conforms to the paradox of enrichment representing a press perturbation (Abrams & Roth, 1994; Rosenzweig, 1971) and widely verified by further theory (Mougi & Nishimura, 2008; Rall et al., 2008), and in experimental aquatic (Persson et al., 2001) and terrestrial (Meyer et al., 2012) systems.

Additionally, as stated in our second hypothesis, the system response varied considerably depending on the moment of perturbation, that is, the point on the attractor at which the perturbation occurred. In particular, we could distinguish two neighboring zones on the attractor which were either the most or the least affected (Figure 7). This difference arose from the intermediate species’ ability to keep the basal level under sufficient top‐down control. This corresponds to previous theoretical (Rall et al., 2008) and experimental findings (Weithoff et al., 2000) in bitrophic food webs, where the authors underlined the importance of the top‐down control to explain the response of systems against a nutrient perturbation. Our tritrophic study also highlights the importance of top‐down processes to dampen the effects of a nutrient pulse and reveals that the top level may strongly affect the response of the food web as a whole (see Appendix 1: A2), because of its decisive influence on the biomasses of the two lower trophic levels (Ceulemans et al., 2021; Wollrab et al., 2012).

By examining different values of the Hill exponent in the basal‐intermediate and intermediate‐top interactions, and different levels of functional diversity, we captured a wide range of dynamical patterns to investigate the relationship between functional diversity and system robustness (recall that robustness is a catch‐all term for resistance, resilience, and elasticity; and see Table 1). In our model, we used Holling type III functional responses (Hill exponent h>1), which are known to dampen dynamics and are also more representative of natural plankton food webs (Uszko et al., 2015). Therefore, in line with previous literature (Rall et al., 2008), we observed that after a nutrient increase a higher Hill exponent dampened the population dynamics. In addition, we showed that the shape (LP and HP states) and type (fixed point, limit cycle or chaotic behavior) of the attractor affected the functional diversity–robustness relationships.

### 4.1 Resistance generally increases with functional diversity

Considering each trophic level as a whole, the web was generally more resistant, as stated in our third hypothesis, since the biomasses did not reach as low values as in the chain (Figures 4 and 6). However, at the population level, the undefended basal species ($B^{u}$) might be more affected in the web than the only basal species in the chain (Figures 4 and 7f, i). In other words, the system's resistance varies with the organization level studied, that is, the trophic or population level.

Under the extremely nutrient‐rich conditions immediately following the perturbation, $B^{u}$ was at a competitive advantage due to its higher growth rate, which explained its dominance during the basal biomass peak over the entire trophic level. As a consequence, the main consumers of $B^{u}$ strongly increased, which, in turn, led to $B^{u}$ being grazed down to very low biomasses. On the other hand, because of its slower growth rate and competitiveness the basal defended species, $B^{d}$, only experienced limited additional growth, and thus only contributed little to the growth of its intermediate consumers. In turn, $B^{d}$ was grazed down less thanks to its lower growth rate and prevented the trophic level from extinction (Figure 7i, l). Therefore, we highlight how a defended (slow‐growing) species in a tritrophic food web increases the resistance of the corresponding trophic level. This is in line with previous studies considering defended species in tritrophic food chains (Loeuille & Loreau, 2004) or slow‐growing plant species (Oliver et al., 2015).

In this way, our model reveals the mechanism behind how species’ functional traits determine their dynamics in a food web. This leads to explicit manifestations of the insurance hypothesis (Naeem & Li, 1997), since a higher resistance is observed at the aggregated trophic level for the web than for the chain, thanks to the average response of different species.

### 4.2 Nutrient pulse pushes system to low‐production state affecting the system's resilience

The resilience of a system can be affected by extinctions, or by the presence of alternative stable states in which all species coexist. Our results showed that the resilience of both the web and the chain strongly depended on the moment of perturbation (Figure 8).

We found that extinction of a complete trophic level was protected by functional diversity; however, extinction of a single species was equally probable in the chain as in the web (Figure 4). The crucial difference was that species extinction in the chain necessarily caused the secondary extinctions of all species at higher trophic levels. In contrast, after an extinction of a single species in the web, much of the trophic structure persisted due to its functional redundancy (Borrvall et al., 2000; Fonseca & Ganade, 2001). Generally, we showed that species extinction occurred regularly in the chain and in the web for perturbation sizes over 10^3^ µg N/L. However, even for much smaller perturbation sizes, both food chain and food web might be vulnerable to a regime shift when the perturbation caused the system to settle on another coexisting attractor.

In our model, both the chain and the web exhibited bistability for a large part of the parameter space (Table 1). In these cases, the system could switch to either the low‐production (LP) or high‐production (HP) state, depending on the initial conditions. This implies that a sufficiently large perturbation could result in the system settling on the other state, which affected its resilience. Such behavior, commonly called a regime shift, is a widely observed phenomenon that can occur in many different types of ecosystems (Folke et al., 2004; Scheffer & Carpenter, 2003). Regime shifts are often the cause of major concern, because the two states may vary considerably in their ecological properties. Some examples are changes in vegetation patterns (Bestelmeyer et al., 2015; Dublin et al., 1990) or transitions between a clear and a turbid state in lakes (Scheffer et al., 1993; Scheffer & Jeppesen, 2007).

In Ceulemans et al. (2019), diversity loss likely caused the system to transition from the HP state to the ecologically undesirable LP state, where top level biomass was much lower, and the biomass dynamics more variable. Our present study revealed that the system is also more likely to transition to the LP than to the HP attractor, when exposed to a sudden nutrient pulse (Figure 8). In particular, we observed that a nutrient pulse could cause the system to behave like the LP state, and a relatively small disturbance of approximately $N_{0}$ (the normal inflow nutrient concentration), or less, could force the system to stay permanently in this state. In addition, this study goes beyond previous work by highlighting the direct and indirect effects of functional diversity on resilience. Functional diversity directly reduces the likelihood of an entire trophic level going extinct, as well as the occurrence of harmful regime shifts; however, it also indirectly enhances the likelihood of such regime shifts (see Figure 3).

Combined with our knowledge of resistance, we highlight the key role played by functional diversity in governing the response of a food web. When functional diversity is high, the HP state persists. The high top biomass level then ensures adequate control on the intermediate level, in turn protecting the basal level from over‐exploitation. However, promoting the probability of being on the HP state increases the risk of a regime shift after a perturbation. A reduction in functional diversity can also abruptly affect food web resilience, as the system is more easily kicked to the LP attractor, where top level biomass is low and the likelihood of trophic levels extinction higher (Figure 3).

### 4.3 Elasticity depends on attractor type, shape, and diversity

Another way to quantify a system's response after a perturbation was by measuring its elasticity, that is, the time it took to return to the pre‐perturbation state (return time, cf. Figure 2). In an economical context, elasticity is an important quantity, because low elasticity means that the desired functioning of an ecosystem may be interrupted for a substantial period of time before returning back to normal (Oliver et al., 2015).

**FIGURE A8:**
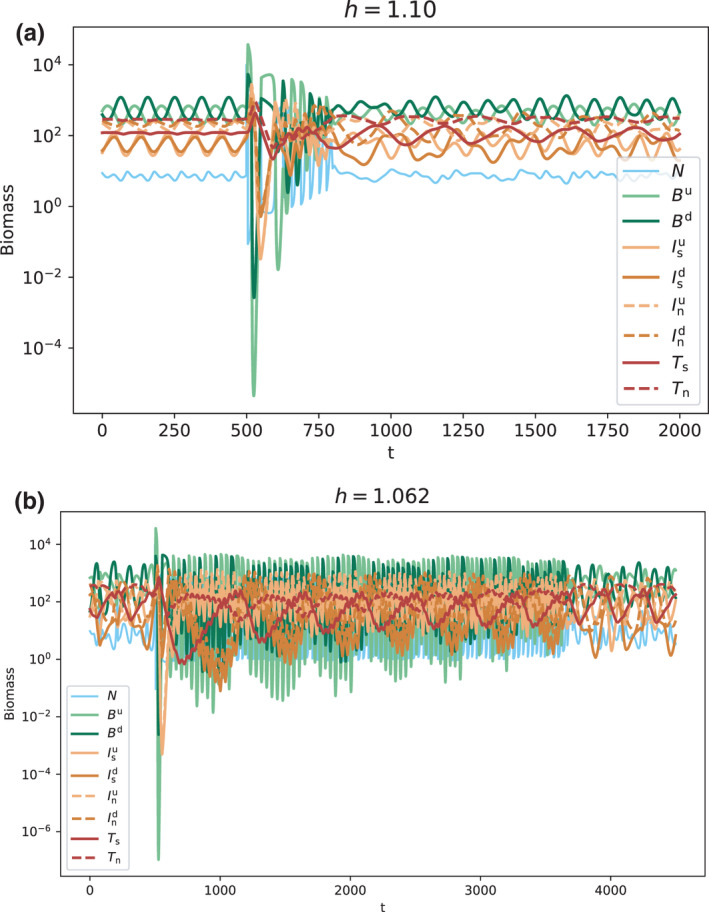
Ghost attractor phenomenon in the post‐perturbation transient. When h=1.10, HP was the only stable state in the food web (cf. Figure A4 and Table 1). However, when perturbed by a nutrient pulse (here $N_{P}=10^{4}$ µg N/L at t=500), the dynamics temporarily behaved like the LP state (see also Figure 7). After approx. 250 time units, the dynamics shifted again to that of the HP state, but required a long time to settle down to regular oscillations. The LP state became unstable for approx. h>1.06 (Ceulemans et al., 2019). Close to this threshold, the dynamics might be on the ghost state for much longer after the same perturbation (note the different time scales in the plots)

Our results showed that the return time increased with the size of the nutrient pulse, and, moreover, this increase could happen in discrete jumps: Suddenly, the dynamics required almost an additional complete cycle before being close again to the attractor (Figures 2 and 9). We also observed that the return time depended on functional diversity and the shape of the attractor to which the perturbed system was returning. In particular, perturbing the HP state could lead to the dynamics permanently (cf. Figure 8) or temporarily (cf. Figures 7 and A8) behaving like the LP attractor. Notably, this could happen even when the LP state was not a dynamical attractor (cf. Figure A8). In this case, the time spent by the transient on this ghost attractor increased with the distance to the bifurcation (Hastings et al., 2018; Morozov et al., 2020), ultimately strongly affecting the return time of the HP state as the Hill exponent decreased.

Notwithstanding the complexities arising from the diversity of attractor types (fixed point, limit cycle, chaotic behavior) and shapes (LP and HP states) as well as the lack of a method to estimate the return time of chaotic attractors, our results suggested a high contextuality of the relationship between functional diversity and elasticity (Figure 9). The absence of clear patterns is in line with previous evidence; for example, a positive relationship between elasticity and functional diversity was observed in empirical studies considering only one trophic level (Schmitt et al., 2020; Smith et al., 2016) whereas a negative relationship was derived from theoretical considerations (Ives & Carpenter, 2007).

Altogether, understanding the effects of functional diversity on robustness, that is, resistance, resilience, and elasticity, and comparing meaningfully food webs with different levels of functional diversity thus require precise knowledge about the systems’ attractor(s). This observation highlights the difficulties in generalizing the diversity–stability relationship: In realistic ecological systems, this relationship and its corresponding mechanisms may become highly complex (Ives & Carpenter, 2007; Loreau & Mazancourt, 2013; McCann, 2000). Depending on a large set of conditions—like the study scale, the habitat, the system complexity, the study type (field, experiment, theory)—previous studies established a positive, neutral, or negative relationship. Therefore, following the advise given by Ives and Carpenter (2007), we put efforts into looking at systems with a wide range of dynamical behaviors and understanding the mechanisms at play. Interestingly, in our systems functional diversity increases the probability of being on the more resistant HP state. However, the low resilience of the HP state, together with its low temporal variability of the biomasses (and vice versa for the LP state), may explain cases of lower elasticity for the HP state, in particular when the Hill exponent is low. Hence, this means that our model explicitly shows how different aspects of stability may co‐vary with each other: Being very stable in one aspect may come at the cost of lower stability in another aspect (see Figure 3; Domínguez‐García et al., 2019; Donohue et al., 2016). Thus, this study highlights the importance of considering more than a single aspect of stability and how they are correlated (Hillebrand & Kunze, 2020; Hillebrand et al., 2018). Overall, despite the distinct structure and parametrization of our model, its general behavior is rather independent of the parametrization (Ceulemans et al., 2019) and its structure is representative for many tritrophic systems as are the fundamental mechanisms at play. This leads us to the expectation that our findings, in particular about functional diversity–stability relationships, are relevant for numerous other systems as well.

## 5 CONCLUDING REMARKS

Overall, this study reveals that tritrophic systems are more strongly affected by a pulse perturbation of higher magnitude and that the moment when the perturbation occurs determines the consequences for the following dynamics of the system. Importantly, we show how functional diversity buffers the effects of a perturbation: Increased functional diversity leads to a higher resistance, resilience, and potentially elasticity and dampens the risk of inducing a regime shift toward an ecologically less desirable stable state. Even though a nutrient pulse only directly affects the basal trophic level, we reveal how top‐down regulatory processes determine the system response. We thus uncover the role of both horizontal (i.e., functional diversity within each trophic level) and vertical diversity (i.e., the number of trophic levels) in governing how food webs respond to disturbances. In this way, the potentially destructive positive feedback loop is mechanistically understood: A loss in functional diversity affects food web functioning in such a way that its resilience, resistance, and elasticity become generally lower, making the food web even more vulnerable to future perturbations.

## CONFLICT OF INTEREST

The authors declare no competing interests.

## AUTHOR CONTRIBUTIONS

**Laurie Anne Wojcik:** Data curation (equal); Formal analysis (equal); Investigation (equal); Methodology (supporting); Resources (equal); Software (equal); Validation (equal); Visualization (equal); Writing‐original draft (equal); Writing‐review & editing (equal). **Ruben Ceulemans:** Conceptualization (equal); Data curation (equal); Formal analysis (equal); Investigation (equal); Methodology (lead); Resources (equal); Software (equal); Validation (equal); Visualization (equal); Writing‐original draft (equal); Writing‐review & editing (equal). **Ursula Gaedke:** Conceptualization (equal); Formal analysis (supporting); Funding acquisition (lead); Investigation (supporting); Project administration (lead); Resources (equal); Supervision (equal); Validation (equal); Visualization (supporting); Writing‐review & editing (supporting).

## Figures and Tables

**FIGURE A1:**
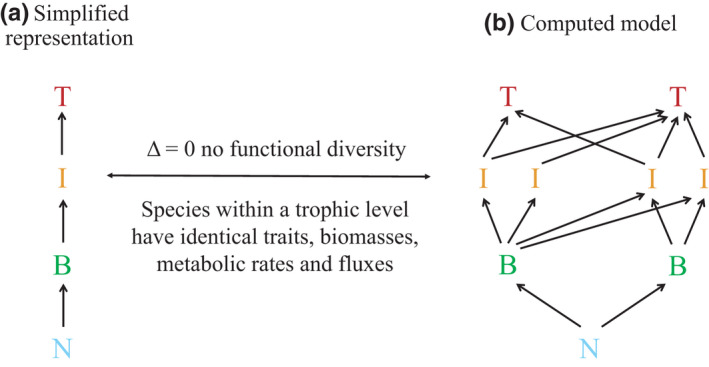
Comparison of two systems with no functional diversity (Δ=0): (a) a food chain with 3 species, and (b) a food web with 8 species where all species at each trophic level have identical traits, biomasses, metabolic rates, and fluxes. These two systems are equivalent with a slight change in the half‐saturation constants. Simulations and analyses were done using the representation (b) but for clarity the representation (a), that is, a chain, is used in the text

**FIGURE A2:**
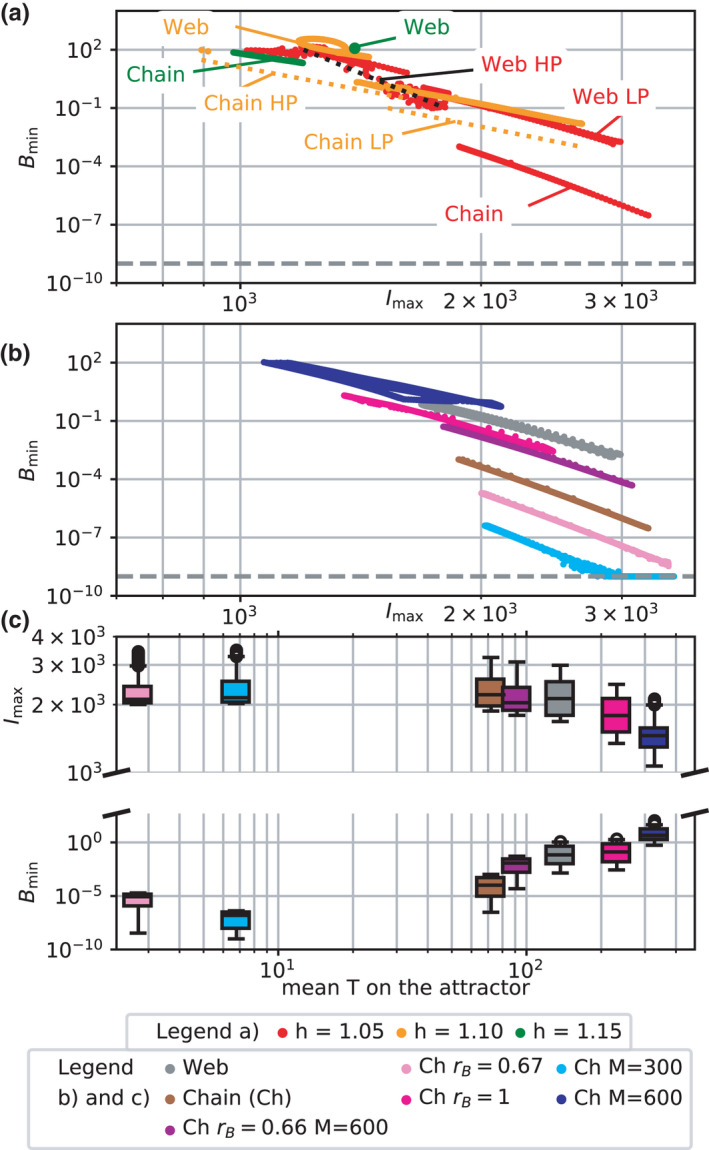
Relationship between the minimal biomass reached by the basal level ($B_{\text{min}}$) and the maximal biomass reached by the intermediate level ($I_{\text{max}}$) after a nutrient pulse perturbation $N_{p}=1000$ µg N/L, depending on where on the attractor the perturbation was applied. The collections of points in the graph are grouped per attractor (LP: low‐production state, HP: high‐production state, cf. Table 1). The dashed gray line at $B_{\text{min}}=10^{‐9}$ µg C/L in the two top panels represents the extinction threshold. A general negative correlation between $B_{\text{min}}$ and $I_{\text{max}}$ was observed: The higher the intermediate level was able to grow after the perturbation, the lower it would graze down the basal level. As expected, this relationship became more pronounced for lower Hill exponents, as the amplitude of the dynamics increased (panel a). Importantly, when comparing the food web to the food chain while keeping the Hill exponent constant, the same $I_{\text{max}}$ led to a higher $B_{\text{min}}$ in the food web. In panels b and c, the parametrization of the food chain varies. The growth rate of only the basal species $r_{B}$ and/or the B‐I half‐saturation constant M were set to different values than their standard values (standard chain values $r_{c}^{\prime }\approx 0.81$ /d and $M_{c}\approx 424$ µg C/L, see Table A1). The relationship between $B_{\text{min}}$ and $I_{\text{max}}$ was influenced by these two traits (panel b) as well as by the biomass of the top trophic level prior to the perturbation (panel c). However, $B_{\text{min}}$ remained generally higher for a given $I_{\text{max}}$ in the web than in the differently parametrized food chains, which shows that functional diversity by itself promoted resistance

**FIGURE A3:**
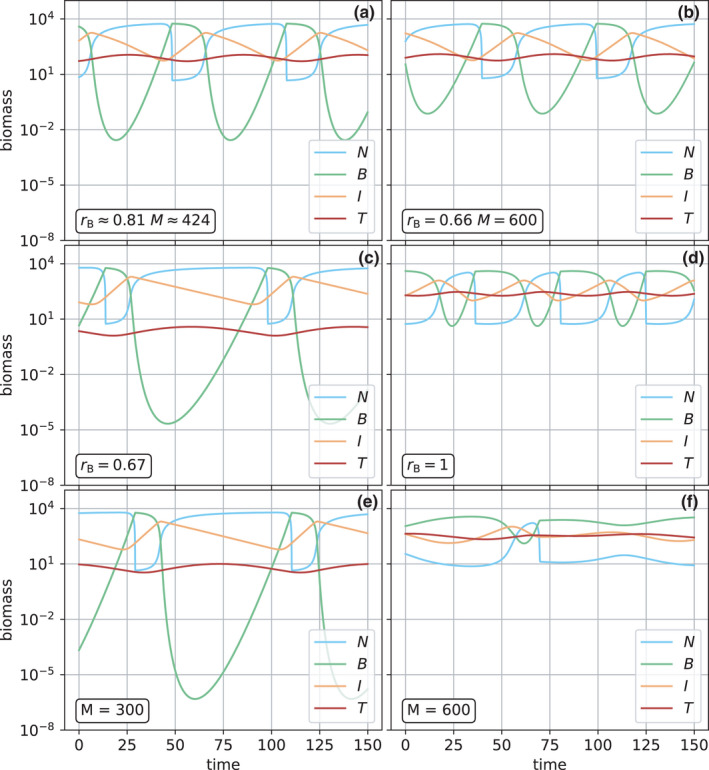
Timeseries of the dynamics on the attractor for the differently parametrized food chains in Figure A2. Panel a shows the standard parametrization of the chain where $r_{B}$and M are logarithmic in‐between the defended and undefended growth rate, and selective and non‐selective half‐saturation constant, respectively. Panels c and d show how the dynamics were affected by setting $r_{B}$ to that of the defended basal species (c) and undefended basal species (d). The change in basal biomass production directly translated to a change in biomass on the top level, which in turn changed top‐down control on the intermediate level. Panels e and f show how changes to the basal‐intermediate half‐saturation constant M had a similar effect on the top level and thus indirectly also on the basal level

**FIGURE A7:**
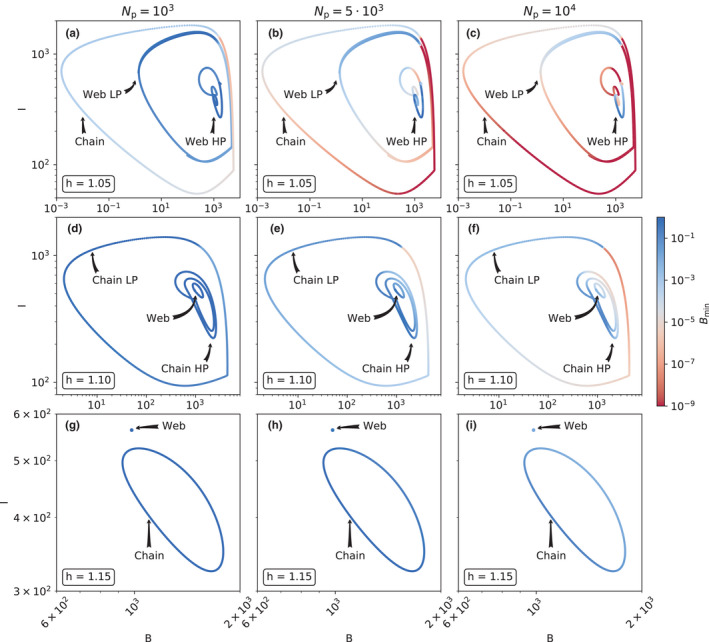
Biomass minima reached by the basal species after a perturbation, $B_{\text{min}}$ depending on its location on the attractor, for different perturbation sizes. The general pattern that the web and LP state resistance tended to be higher than the chain and HP state, respectively, held for different perturbation sizes increasing from right to left and different Hill exponents increasing from top to bottom

## Data Availability

The code and data required to produce and analyze the results are available on Dryad https://doi.org/10.5061/dryad.qrfj6q5h3.

## APPENDIX 1

### A1 Additional information about the model

#### A1.1 Parameter description and values

**TABLE A1:** Parameter values used as described in the tritrophic chemostat model developed by Ceulemans et al. (2019)

Parameter name	Parameter value
Body mass ratio between adjacent TLs	*m_I_*/*m_B_* = *m_T_*/*m_I_* = 10^3^
Allometric scaling exponent	λ=‐0.15
Inflow nutrient concentration (μg N/L)	$N_{0}=1120$
Dilution rate	δ=0.055
Nutrient half‐saturation const. of *B* (μg N/L)	$h_{N}=10$
Nitrogen‐to‐carbon ratio of *B*	*c_N_*/*c_C_* ≈0.175
Conversion efficiency	e=0.33
*T* max. grazing rate (per day)	$\gamma^{\prime }\approx 0.38$
Hill exponent	$h=\left\{1.05,1.10,1.15\right\}$

Parameter name	Chain	Web
*B* max. growth rate (per day)	$r_{c}^{\prime }\approx 0.81$	$r_{u}^{\prime }=1$, $r_{d}^{\prime }=0.66$
*I* max. grazing rate (per day)	$g_{c}^{\prime }\approx 0.87$	$g_{u}^{\prime }\approx 1.08$, $g_{d}^{\prime }\approx 0.71$
*B*–*I* half‐saturation const. (μg C/L)	$M_{c}\approx 424$	$M_{s}=300$, $M_{n}=600$
*I*–*T* half‐saturation const. (μgC/L)	$\mu_{c}\approx 424$	$\mu_{s}=300$, $\mu_{n}=600$

The chain and the web have different parametrizations according to the trade‐offs between the relevant traits. As a consequence, the B and I maximal growth rates, as well as the B‐I and I‐T half‐saturation constant decrease with the defense for the prey and the selectivity for the predators, respectively. The trait values of the standard chain are included in the range of the values set for the web, such that $r_{u}^{\prime }>r_{c}^{\prime }>r_{d}^{\prime }$, $g_{u}^{\prime }>g_{c}^{\prime }>g_{d}^{\prime }$, $M_{s}>M_{c}>M_{n}$, and $\mu_{s}>\mu_{c}>\mu_{n}$. Simulations of the chain and the web are compared for three different values of the Hill exponent.

#### Food chain

Following Ceulemans et al. (2019), the food chain was modeled as a food web where species at each trophic level had identical traits, such that there was no functional diversity within each trophic level (see Figure A1 for the topology). The exact trait values in the chain ($\theta_{c}$) were calculated from those in the food web as follows:

(A1)
$$\text{log}\;\theta_{c}=\frac{\text{log}\;\theta_{a}+\text{log}\;\theta_{b}}{2},$$

where a=u and b=d for $\theta =r^{\prime }$ or $\theta =g^{\prime }$; or a=s and b=n for θ=M or θ=μ.

#### Food web

In the functionally diverse food web, the trait differences between the species on every trophic level were controlled by the trait difference parameter Δ. When Δ=0, the species had identical trait values (food chain), whereas when Δ=1, the trait differences were maximal. The parameter values varied logarithmically with functional diversity in such a way that parameter changes were proportional to the centered trait value (corresponding to the parametrization of the food chain) in both directions, since the basal maximal growth rate ($r_{i}^{\prime }$) and the intermediate grazing rates ($g_{i}^{\prime }$) appeared as linear factors in the differential equations. We used this method consistently for all the parameters varying with functional diversity. For a detailed description of how the trait values varied with Δ, see Ceulemans et al. (2019).

#### Equivalency between food chain and food web when ∆ = 0

In this section, we briefly motivate why a chain with four elements and a food web with nine elements and Δ=0 are fully comparable, such that we did not introduce a bias when studying the effect of altered functional diversity by seemingly changing the system's dimensionality.

When the trait values of all species at the same trophic level are identical, the systems’ equations (Equation 2) can be rewritten by introducing new state variables B, I, T, such that:

(A2)
$$B=B_{1}+B_{2}\;I=I_{1}+I_{2}+I_{3}+I_{4}\;T=T_{1}+T_{2}.$$

When doing so, the equations for a four‐dimensional food chain are almost exactly recovered besides a slight change in the half‐saturation constants of the functional responses:

(A3)
$$M_{c}\to 2^{\left(h‐1\right)/h}M\;\text{and}\;\mu_{c}\to 4^{\left(h‐1\right)/h}\mu ,$$

where $M_{c}$ and $\mu_{c}$ are the half‐saturation constants of the four‐dimensional equivalent of the nine‐dimensional food web with Δ=0, and M and μ are the half‐saturation constants of the nine‐dimensional food web. In addition, since the Hill exponent values in this study are close to 1 ($h\in \left\{1.05,1.10,1.15\right\}$), the difference is small even for the highest Hill exponent h=1.15: $M_{c}\to 1.09M$ and $\mu_{c}\to 1.2\mu$. A precise mathematical argument of this equivalency is presented in the supplementary information of Ceulemans et al. (2019).

#### A2 The *B*_min_–*I*_max_ relationship

In this section, we provide more details about the relationship between the minimal basal biomass ($B_{\text{min}}$) and the maximal intermediate biomass ($I_{\text{max}}$) reached after the perturbation, that we describe in the main text. We verified that this negative relationship held for different parametrizations (Hill exponent h, basal growth rate $r_{B}$—this notation indicates that only the basal growth rate is changed rather than the growth rates of all species as they are allometrically scaled in the model—and intermediate half‐saturation constant M). Particularly, we investigated how functional diversity influenced this $B_{\text{min}}‐I_{\text{max}}$ relationship by comparing the food web alongside differently parametrized food chains, and we also uncovered the principal role of the top trophic level in governing the response to a nutrient pulse perturbation in tritrophic systems (Figure A2).

The negative relationship between $B_{\text{min}}$ and $I_{\text{max}}$ was influenced by several food web parameters, such as the Hill exponent (Figure A2a): Lowering the Hill exponent increased the oscillation amplitude of the dynamics, such that systems with h=1.05 at the low‐production (LP) state obtained the lowest $B_{\text{min}}$ values. Recall that the Hill exponent determined the attractor shape and type (see Table 1). In particular, the probability of being on the high‐production (HP) state increased for higher Hill exponent values. We also observed that the HP state had lower $I_{\text{max}}$ and higher $B_{\text{min}}$ values, as compared to the LP state (Figure A2a). Because the HP state was characterized by a higher top species biomass and smaller amplitudes (Figure 7a and d), it did not reach the extreme values of the LP state (Figure 7c and f). Moreover, the top species kept the intermediate species under stronger top‐down control, which limited their response to a nutrient pulse. Therefore, this analysis of the relationship between $B_{\text{min}}$ and $I_{\text{max}}$ confirms that increasing the Hill exponent has a stabilizing effect and that the HP state has a higher resistance than the LP state.

Importantly, our results showed that $B_{\text{min}}$ tended to be higher in the food web than in the food chain (Figure A2a). A given $I_{\text{max}}$ led to a higher $B_{\text{min}}$ in the food web (insofar as they can be compared). This suggests that a more diverse food web is more resistant to a pulse perturbation than a food chain with little or no diversity.

To untangle a potential direct effect of diversity on the $B_{\text{min}}‐I_{\text{max}}$ relationship from the effect of comparing basal and intermediate species with different growth rates and interaction parameters in the chain and web, we also compared the food web to differently parametrized food chains (Figure A2b). The maximal basal growth rate in the food chain ($r_{c}^{\prime }\approx 0.81$ /d) lay in‐between the defended basal growth rate ($r_{d}^{\prime }=0.66$ /d) and the undefended basal growth rate ($r_{u}^{\prime }=1$ /d) of the more diverse food web. Additionally, the basal‐intermediate half‐saturation constant in the food chain ($M_{c}\approx 424$ µg C/L) also lay in‐between the values of the basal‐selective ($M_{s}=300$ µg C/L) and basal non‐selective ($M_{n}=600$ µg C/L) interactions of the more diverse food web. Evidently, changing these parameters in the food chain also modified the resulting $B_{\text{min}}‐I_{\text{max}}$ relationship, either through direct (such as grazing suppression at low prey densities when M is high) or indirect effect (such as differing top biomasses when the basal growth rate $r_{B}$ was changed; Figure A2c).

Importantly, in the food web, the growth rates of the undefended and defended I species differed from the growth rate of I in the food chain. However, in neither a chain where all growth rates were scaled to reflect that of the defended species, nor of the undefended species, it was possible for the top trophic level to survive. We therefore compared the food web to a chain where only the basal growth rate was altered. In the food chain with $r_{B}$ set to the high value of the undefended species in the web (*B^u^*), $B_{\text{min}}$ remained approximately one order of magnitude below $B_{\text{min}}$ of the food web, whereas reducing $r_{B}$ to the value of the defended species (*B^d^*) decreased $B_{\text{min}}$ by approximately two orders of magnitude below $B_{\text{min}}$ of the standard chain (Figure A2b). These changes are due to strong differences in the mean biomass on the top level in the alternative food chains: when $r_{B}$ was high, the increased basal productivity translated to an increased biomass in the top level, and vice versa (cf. Figures A2c and A3). When the top biomass was higher, stronger grazing pressure on the intermediate level prevented excessive grazing of B. On the other hand, for low top biomass the intermediate level could remain at high biomass for an extended period of time, until they found no more food.

Similarly, increasing the B‐I half‐saturation constant in the chain to the value of the non‐selective consumers in the web ($M_{n}=600$ µg C/L) increased $B_{\text{min}}$, and decreasing M to the value of the selective consumers in the web ($M_{s}=300$ µg C/L) correspondingly decreased $B_{\text{min}}$. These changes were caused by the grazing suppression at low B densities, when M was increased. However, the changed mean top biomass caused by the altered basal productivity dominated the changes in $B_{\text{min}}$ when varying M (cf. Figures A2c and A3).

In the food web, $B^{u}$ was generally grazed to lower densities than $B^{d}$ (Figure 7h, i). This means that the $B^{d}‐I_{n}$ interaction (low r, high M) was principally responsible for the value of $B_{\text{min}}$ (Figure A2b). Remarkably, in a chain parametrized with such a low $r_{B}$ and high M, $B_{\text{min}}$ was still approximately one order of magnitude below that of the food web. In summary, functional diversity generally enhanced the system's resistance and this held true after accounting for the fact that the negative relationship between $B_{\text{min}}$ and $I_{\text{max}}$ was influenced by several factors (Hill exponent, basal growth rate, basal‐intermediate half‐saturation constant, and top biomass).
